# Identification of multiple proteoforms biomarkers on clinical samples by routine Top-Down approaches

**DOI:** 10.1016/j.dib.2018.03.114

**Published:** 2018-03-31

**Authors:** Jerome Vialaret, Pierre-Olivier Schmit, Sylvain Lehmann, Audrey Gabelle, Jason Wood, Marshall Bern, Rainer Paape, Detlev Suckau, Gary Kruppa, Christophe Hirtz

**Affiliations:** a University of Montpellier, LBPC, IRMB, CHU de Montpellier, 34000 Montpellier, France; b Bruker Daltonique, S.A, 34, rue de l’industrie, 67160 Wissembourg, France; c Centre Mémoire Ressources Recherche, CHU Montpellier, hôpital Gui de Chauliac, Université Montpellier I, Montpellier F-34000, France; d Bruker Daltonics Inc., 40 Manning Road, Billerica, MA 01821, USA; e Protein Metrics Inc., 1622 San Carlos Ave., San Carlos, CA 94070 USA; f Bruker Daltonik GmbH, Fahrenheitstrasse 4, 28359 Bremen, Germany

**Keywords:** Top-Down label-free proteoform profiling, Clinical proteomics, Alzheimer disease, Ultra-high resolution Q-Tof

## Abstract

Top-Down approaches have an extremely high biological relevance, especially when it comes to biomarker discovery, but the necessary pre-fractionation constraints are not easily compatible with the robustness requirements and the size of clinical sample cohorts. We have demonstrated that intact protein profiling studies could be run on UHR-Q-ToF with limited pre-fractionation (Schmit et al., 2017) [1]. The dataset presented herein is an extension of this research.

Proteoforms known to play a role in the pathophysiology process of Alzheimer's disease were identified as candidate biomarkers. In this article, mass spectrometry performance of these candidates are demonstrated.

**Specifications Table**

**Table:** 

Subject area	*Biology*
More specific subject area	*Clinical chemistry, Biomarker analysis, proteoform profiling*
Type of data	*Tables, figures*
How data was acquired	*Ultimate nano-RSLC system (Thermo Fischer Scientific Waltham, USA) coupled to Impact II™ benchtop UHR-Q-ToF (Bruker Daltonik, Bremen, Germany) through a CaptiveSpray nanoBooster*™ *source (Bruker Datonik, Bremen, Germany)*
Data format	*Analyzed and processed data*
Experimental factors	*CSF was collected in polypropylene tubes under standardized conditions between 9 *a.m. *and 1 *p.m. *in order to minimize the effects of diurnal variations. Each CSF sample was sent within 4 *h *of being collected to the local laboratory, where it was centrifuged at 1000* × *g for 10 *min *at a temperature of 4 *°C*. CSF was then aliquoted into 1.5 *ml *polypropylene tubes and stored at −80* °C*.*
Experimental features	*500 *µL *of human CSF mixed with twenty-five microliters of 70% perchloric acid, added for protein precipitation. Supernatants were collected and protein clean-up was performed using Oasis HLB µelution well plates. Eluted proteins were dried and resuspended with A phase before LCMS analysis.*
Data source location	*IRMB, Montpellier hospital, France*
Data accessibility	*Data is with article*

**Value of data**

• Proof of concept for intact protein analysis on biofluid.
• This data exhibited identified proteoforms originating from CSF of Alzheimer's disease patients
• Proteoform sequences and/or modifications will be shared with the community to extend available information, in order to better understand the physiopathology.

## 1 Data

Proteins contained in CSF were directly analyzed by LCMS with a Top-Down approach. This type of analysis gave information of proteoforms composition. This proof-of-concept study was applied to a patient cohort (30 samples) in Alzheimer's disease context. These samples were separated into 3 groups: group 1 (patients with Alzheimer's disease), group 2 (patients with other neurodegenerative diseases), and group 3 (patients with non-neurodegenerative diseases).

The number of compounds after MS analysis (filtered for charge state >1 and SNAP correlation >0.75, compounds with mass difference of less than 2 ppm and retention time differences of less than 2 min were considered as identical) totaled between 12,000 and 18,000. More than 5000 compounds common to the datasets from all 30 patients were used for the statistical analysis. Compounds whose *p*-value was below a 0.02 threshold were then tested for correlation (Pearson's correlation) with all the clinical markers including AD markers (Tau concentration; results of memory tests). Positively correlated compounds (*r*^2^>0.8) were then selected for further MS/MS analysis and their identifications. MS results containing monoisotopic pattern, Extraction Ion Chromatogram, and MS/MS spectra were presented in 6 figures.

Proteoforms found to be regulated in AD pathology are listed in Table 1. These proteoforms come from 3 canonical proteins: clusterin, secretogranin-2, or chromogranin-A. These proteins were known as biomarkers of AD and neurodegenerative disorders [1].

**Fig. 1:**
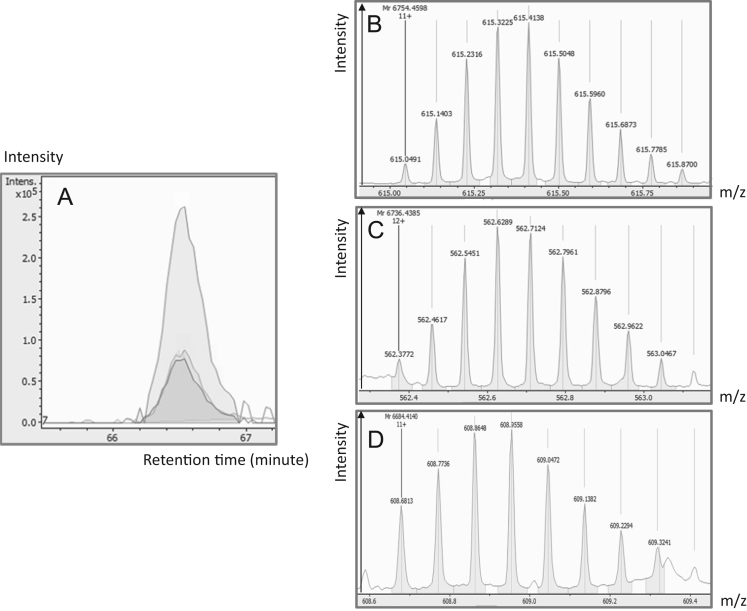
Clusterin proteoforms identification (CLUS-01, CLUS-02, CLUS-03). A: Extracted Ion Chromatogram of 615.0491*m*/*z* (CLUS-01), 562.3772*m*/*z* (CLUS-02), 608.6813*m*/*z* (CLUS-03). B: Monoisotopic pattern of CLUS-01 on MS1 spectra. C: Monoisotopic pattern of CLUS-02 on MS1 spectra. D: Monoisotopic pattern of CLUS-03 on MS1 spectra.

**Fig. 2:**
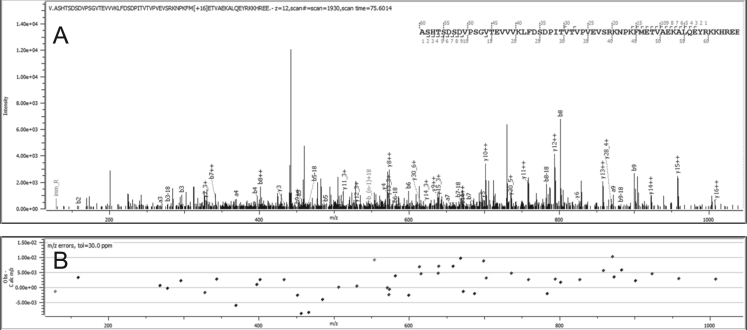
Clusterin proteoform Clus-01 identification on MS/MS spectra with Byonic™. A: Annotated MS/MS spectra by Byonic™. B: Mass error observed on each match ion.

**Fig. 3:**
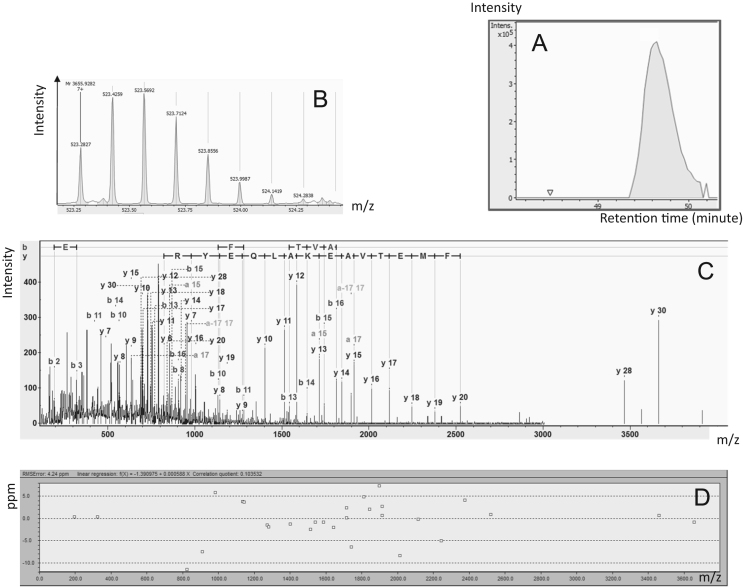
Clusterin proteoforms identification (CLUS-04). A: Extracted Ion Chromatogram of 523.2827*m*/*z*. B: Monoisotopic pattern of CLUS-04 on MS1 spectra. C: Annotated MS/MS spectra by Mascot. D: Mass error observed on each match ion.
– Secretogranin-2 proteoforms are shown in Fig. 4. One proteoform corresponding to the middle part (182–214) of the protein was identified by Byonic™ with a score of 1213.1.

– Clusterin proteoforms are shown in Fig. 1, Fig. 2, Fig. 3.
  CLUS-01 to 03 present C termini part of the full-length protein starting at position 390 or 391 (Fig. 1). These closed species co-eluted. CLUS-01 was identified by Byonic™ (Fig. 2) with a score of 770.1, and the two-other species were eluted by mass differences (below 2.4 ppm) on MS1 level. CLUS-04, a shorter proteoform starting at position 420 was identified by Mascot with a peptide score of 52, with a good mass precision (MS1: 3.4 ppm; MS2: 4.24 ppm) (Fig. 3).

**Fig. 4:**
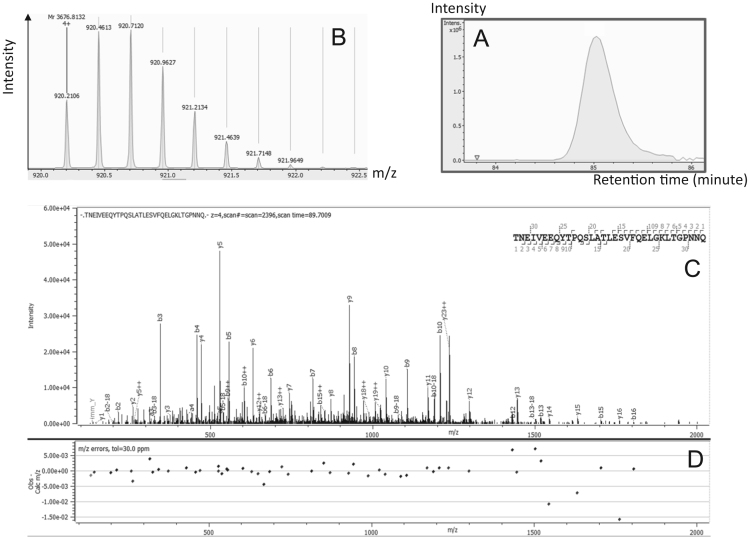
Secretogranin-2 proteoform identification. A: Extracted Ion Chromatogram of 920.2106*m*/*z*. B: Monoisotopic pattern of Secretogranin-2 proteoform on MS1 spectra. C: Annotated MS/MS spectra by Byonic™. D: Mass error observed on each match ion.
– Chromogranin-A proteoforms were show on Fig. 5, Fig. 6. Two proteoforms were detected (Chrom-01 and Chrom-02). These proteoforms were detected with very different intensities that indicated a completely different stoichiometry. Form 439 to 457 was present in very low quantity and MS/MS identification required manual *de novo* sequencing. This sequencing used very high criteria in term of mass precision at MS1 level (<3 ppm) and MS2 level (<10 ppm). A longer proteoform based on MS1 ion extraction was 37.5 times higher and could be identified with Byonic™ with a score of 468.2 (Fig. 6).

**Fig. 5:**
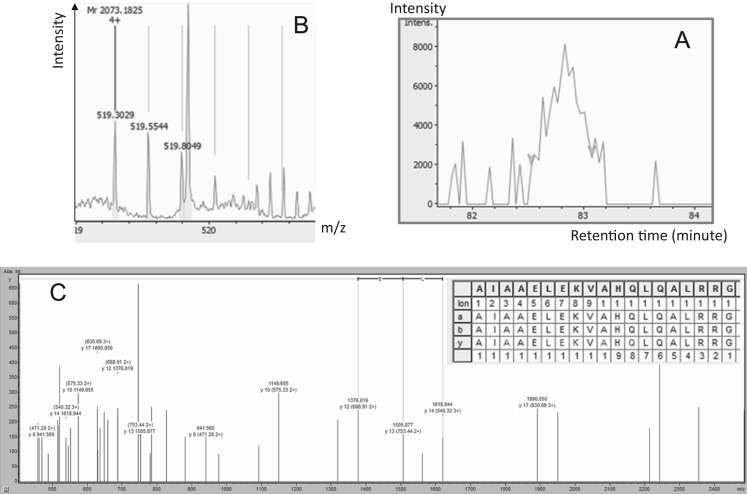
Chromogranin-A proteoform identification (Chrom-01). A: Extracted Ion Chromatogram of 519.3029*m*/*z*. B: Monoisotopic pattern of Chrom-01 on MS1 spectra. C: Annotated MS/MS spectra identified manually with BioTools 3.2. MSMS mass tolerance below 10 ppm was used.

**Fig. 6:**
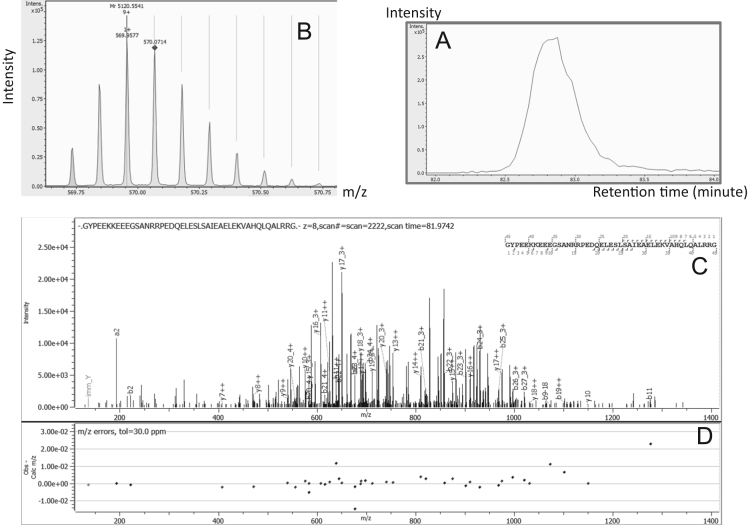
Chromogranin-A proteoform identification (Chrom-02). A: Extracted Ion Chromatogram of 569.9577*m*/*z*. B: Monoisotopic pattern of Chrom-02 on MS1 spectra. C: Annotated MS/MS spectra by Byonic™. D: Mass error observed on each match ion.

**Table 1:** Display of proteoforms originating from 3 different canonical proteins.

**ID**	**Identifier and proteoforms**	**Charge (*z*)**	***m*/*z* observed**	**Deconvoluted mass *m*/*z***	**Sequence found**	**PTMs**	**Position in the full sequence**	***p*-Value**	**Retention time (min)**	**Mass error (ppm)**
Clusterin P10909	Clus-01	11	615.0491	6754.46	V.ASHTSDSDVPSGVTEVVVKLFDSDPITVTVPVEVSRKNPKFMETVAEKALQEYRKKHREE.	M42(Oxidation)	390–449 (C ter)	0.004	66.3	0.1
Clusterin P10909	Clus-02	12	562.3772	6736.48	V.ASHTSDSDVPSGVTEVVVKLFDSDPITVTVPVEVSRKNPKFMETVAEKALQEYRKKHREE.		390–449 (C ter)	0.02	66.3	2.4
Clusterin P10909	Clus-03	11	608.6813	6684.44	A.SHTSDSDVPSGVTEVVVKLFDSDPITVTVPVEVSRKNPKFMETVAEKALQEYRKKHREE.	M41(Oxidation)	391–449 (C ter)	0.001	66.3	2.2
Clusterin P10909	Clus-04	7	523.2827	3656.95	V.PVEVSRKNPKFMETVAEKALQEYRKKHREE.		420–449 (C ter)	0.222	49.3	3.4
Secretogranin-2 P13521	SCG2	4	920.2106	3677.82	R.TNEIVEEQYTPQSLATLESVFQELGKLTGPNNQ.K		182–214	0.02	84.9	0.2
Chromogranin-A P10645	Chrom-01	4	519.3029	2073.19	S.AIAAELEKVAHQLQALRRG.	E3->A (Mutation)	439–457	0.06	75.4	2.9
Chromogranin-A P10645	Chrom-02	9	569.9577	5119.57	R.GYPEEKKEEEGSANRRPEDQELESLSAIEAELEKVAHQLQALRRG.		413–457 (C ter)	0.1	77.3	1.4

## 2 Experimental design, materials and methods

Experimental design and the materials and methods have been reported previously [1].

### 2.1 CSF sample preparation

500 μL of human CSF were mixed with twenty-five microliters of 70% perchloric acid (Fluka analytical, Sigma Aldrich) for protein precipitation and samples and kept on ice for 15 min before centrifugation (15 min, 4 °C and 16,000 g). Supernatants were collected and mixed with 50 μL of 1% trifluoroacetic acid (TFA, Sigma, Lisle d’Abeau Chesnes, France). Protein clean-up was performed using Oasis HLB μelution well plates. Cartridge was conditioned with 300 μL of methanol; followed by 500 μL of 0.1% TFA. Acidified samples were loaded onto the plate and washed two times with 500 μL of 0.1% TFA. Retained proteins were eluted with 100 μL acetonitrile/acidified water with 0.1% TFA (35/65 v/v). The eluted sample was dried on a vacuum concentrator (Labconco, USA). Samples were suspended with 25 μL of solvent A before LCMS analysis, vortexed at 1000 rpm for 10 min and transferred on polypropylene vial.

### 2.2 LC-MS high flow and low flow separation

LC-MS High Flow separation and profiling were performed 213 by connecting an Aeris Widepore C4, 150×2.1, 3.6 μm column (Phenomenex, Torrance, USA) to the Loading pump of an Ultimate nanoRSLC system (Thermo Fisher Scientific, Waltham, USA). The flow rate was set to 500 μl/min and the column temperature was set to 50 °C. The solvents used for the elution were MilliQ water containing 1% formic acid (solvent A) and Acetonitrile containing 0.8% formic acid (Solvent B). 15 μl of each sample have been injected and separated with a 45 min method (2% B for 2 min, ramp to 12% in 3 min, then ramp to 30% B in 28.5 min, ramp to 90% B in 2 min, 90% B maintained for 4 min, Ramp down to 2% B in 1.5 min, re-equilibrate for 4 min).The LC-system was coupled to an Impact II benchtop UHR224 Q-TOF (Bruker Daltonik, Bremen, Germany) through the Appolo ESI source. Drying gas flow and temperature were set to 9 l/min and 200 °C, respectively, and nebulizer gas pressure was set to 2.1 bars. MS acquisition rate was set to 2 Hz and data have been acquired over a 300–4000 *m*/*z* mass range. A loop injection of Esi Tuning Mix (Agilent Technologies, Santa Clara, USA) was used to have a calibrant signal recorded at the beginning of every chromatogram.

LC-MS Low Flow separation and profiling were performed on a Proswift RP-4H 50 cm×100 μm monolithic column (Thermo Fisher Scientific, Waltham, USA) after pre-concentration on an Acclaim PepMap300, 5 μm, 300 Å Wide Pore, 300 μm×5 mm C4 cartridge (Thermo Fischer Scientific Waltham, USA). Separation was performed on an Ultimate nano-RSLC system (Thermo Fischer Scientific Waltham, USA). The loading pump flow was set to 30 μl/min and the nanoPump flow was set to 1 μl/min. The column temperature was set to 40 °C. The solvents used for the elution were MilliQ water containing 1% formic acid (solvent A) and acetonitrile containing 0,8% formic acid (Solvent B). The loading solvent was MilliQ water containing 0,1% TFA. TFA was purchased from Sigma (Lisle d’Abeau Chesnes, France) 2 μl of each sample have been injected and separated with a 180 min method (5% B for 5 min, ramp to 9% B in 10 min, then ramp to 35% B in 110 min, ramp to 40% B in 8 min, ramp to 60% B in 9 min and ramp to 95% B in 3 min maintained for 15 min. Ramp down to 5% B in 3 min, re-equilibrate for 23 min). The nano-LC-system was coupled to an Impact II™ benchtop UHR-Q-ToF (Bruker Daltonik, Bremen, Germany) through a CaptiveSpray nanoBooster™ source (Bruker Datonik, Bremen, Germany). Drying gas flow and temperature were set to 5 l/min and 180 °C, respectively, and nanoBooster gas pressure was set to 0.2 bars. The nanoBooster reservoir was filled with acetonitrile.MS acquisition rate was set to 1 Hz and data have been acquired over a 249 300–4000 *m*/*z* mass range. LC-MS/MS acquisitions have been performed with the same LC-MS setup by having the Impact II operated in Auto MS/MS mode with a Scheduled Precursor List (SPL) to target the proteoforms of interest.

### 2.3 LC-MS data processing for CSF samples

Data Processing: LC-MS data were automatically processed (calibration, protein signal extraction with Dissect™, deconvolution and determination of monoisotopic masses with SNAP™, charge state filtering, similarity filtering, export of deconvoluted monoisotopic masses with corresponding retention time and intensities) in Data Analysis 4.2™ (Bruker Daltonik, Bremen, Germany). Singly charged compounds have been automatically excluded. Only the isotopically resolved compounds have been taken into account. Statistical analyses were performed with Profile Analysis 2.1™ (Bruker Daltonics). The retention times, intensities and deconvoluted masses obtained for each compound from the Data Analysis processing have been used to generate the bucket table. The mass accuracy and retention time tolerance were set to 2 ppm and 0.5 (High Flow analysis) or 2 min (Low Flow analysis). Compounds sharing the same mass and retention time coordinates within those tolerances have been considered as similar. The bucket tables were built with all compounds present at least in 60% of one class, and the missing values were replaced by the average value of the bucket in the class the analysis belongs to. Intensities values were then normalized with the quantile normalization algorithm available in Profile Analysis. A student's *t*-test was performed to reveal compounds that were capable of discriminating 2 classes (*p* value <0.02). Statistical analysis was performed with the MedCalc™ 12.1.4.0 software.

### 2.4 LC-MS/MS data processing and identification for CSF samples

LC-MS/MS data were automatically processed (calibration, creation of the LC-MS/MS compound list, deconvolution, export of XML list with deconvoluted parent ion and fragment spectra masses and intensities) in Data Analysis 4.2™ (Bruker Daltonik, Bremen, Germany) Identifications were done either manually with BioTools 3.2™ (Bruker Daltonik, Bremen, Germany) or automatically with Byonic™ (ProteinMetrics, SanCarlos, USA). With BioTools, the Top-Down Sequencing search 285 functionality was used with Mascot 2.4(Matrix Science) to identify proteoforms with a partially unmodified sequence. When this approach did not suffice to identify the protein the designated proteoform originates from, a blast search that was performed after an initial tag determination. In both cases, the full characterization was then obtained by mutation/modification searches performed with the Sequence Editor functionality available in BioTools 3.2™. Byonic searches Top-Down data in the same way as Bottom-Up data, meaning that the user supplies a protein database, allowed PTMs, and specificity of N- and C-termini, where “fully specific”. Byonic searches were performed with various protein databases (one containing only full secretogranin, transthyretin, and chromogranin sequences; modifications are applied to all potential sites in a protein, with separate limits for each type of modification as well as a limit on the total number of modifications. The searches allowed 10 ppm precursor mass tolerance, 30 ppm fragment mass tolerance, and symmetric “narrow” compensation for precursor monoisotopic mass calls, which allows no error in nominal mass for precursors up to 2500 Da, ±1 Da error for precursors from 2500 to 5000 Da, and ± 2 Da error for precursors of mass greater than 5000 Da.
