# Supplementary material for: Identification of multiple proteoforms biomarkers on clinical samples by routine Top-Down approaches
Source: Data Brief. 2018 Mar 31;18:1013–21. doi: 10.1016/j.dib.2018.03.114 (PMC5996497; doi:10.1016/j.dib.2018.03.114)
Supplement: Supplementary file 1 — Supplementary material [file mmc1.doc]

**Conflict of Interest Form**

**Data In Brief**

Corresponding author: Christophe Hirtz ([christophe.hirtz@umontpellier.fr](mailto:christophe.hirtz@umontpellier.fr))

University of Montpellier, CHU Montpellier, Institute of Regenerative Medicine & Biotherapy, 80 Avenue Auguste Fliche, Montpellier, 34925, France. Electronic address: [christophe.hirtz@umontpellier.fr](mailto:christophe.hirtz@umontpellier.fr)

Submission date : 27/11/2017

Identification of multiple proteoforms biomarkers on clinical samples by routine Top-Down approaches

Authors: Jerome Vialaret^1^, Pierre-Olivier Schmit^2^, Sylvain Lehmann^1^, Audrey Gabelle^1,3^, Jason Wood^4^, Marshall Bern^5^, Rainer Paape^6^, Detlev Suckau^6^, Gary Kruppa^4^ and Christophe Hirtz^1^

Affiliations:

1 – University of Montpellier, LBPC, IRMB, CHU de Montpellier, 34000 Montpellier, France

2 - Bruker Daltonique S.A, 34, rue de l’industrie, 67160 Wissembourg, France.

3 - Centre Mémoire Ressources Recherche, CHU Montpellier, hôpital Gui de Chauliac, Montpellier. Université Montpellier I, Montpellier, F-34000 France.

4 - Bruker Daltonics Inc.40 Manning Road, Billerica, MA 01821, USA

5 – Protein Metrics Inc, 1622 San Carlos Ave.,San Carlos, CA 94070 USA

6 – Bruker Daltonik GmbH, Fahrenheitstrasse 4, 28359 Bremen, Germany

I certify that this research is original, not under publication consideration elsewhere, and free of conflict of interest.

**Authorship**

I certify that I am the corresponding author for this manuscript. The manuscript is submitted with the knowledge and on behalf of the listed co-authors.

I certify that each co-author listed above participated sufficiently in the work to take responsibility for the content, and that all those who qualify are listed. Contributors

I certify that no funded writing assistance was utilised in the production of this manuscript.

**Author disclosures**

I certify that none of the authors have any relevant financial and/or nonfinancial relationships to disclose.
